# Genomic Identification and Characterization of the Cotton *YABBY* Gene Family

**DOI:** 10.3390/genes17010064

**Published:** 2026-01-06

**Authors:** Guoqiang Zhang, Zhen Liu, Mingli Xia, Sainan Zhang, Zhixian Li, Quanwei Lu

**Affiliations:** Anyang Key Laboratory of Bioinformatics, School of Biotechnology and Food Engineering, Anyang Institute of Technology, Anyang 455000, China20160513@ayit.edu.cn (Z.L.);

**Keywords:** *YABBY*, cotton, duplication, divergence, expression

## Abstract

**Background**: *YABBY* transcription factors play important roles in plant growth and development. Although this gene family has been characterized in many plant species, a comprehensive analysis in cotton remains unavailable. **Methods**: In this study, we investigated the YABBY gene family in cotton by integrating multiple bioinformatics methods. **Results**: *YABBY* genes were identified in the genomes of four cotton species (*Gossypium hirsutum*, *Gossypium barbadense*, *Gossypium arboretum* and *Gossypium raimondii*) and these identified genes were further classified into six groups. Following this classification, the expansion of the cotton *YABBY* gene family was examined, and we found that the family exhibits diverse expansion patterns during evolution, with segmental duplication acting as the primary driving force. In contrast, the notably larger repertoire of *YABBY* genes in *G. raimondii* is likely attributable to transposon activity. Regarding their evolutionary trajectory, Ka/Ks analysis showed that the *YABBY* gene family has undergone purifying selection. Beyond evolution, expression and cis-element analyses further demonstrated that YABBY genes possess diverse functions. In addition, we identified *YABBY* genes involved in different developmental stages of cotton fibers. **Conclusions**: We clarify the function and evolution of the cotton *YABBY* gene family in this work, and these results will provide a critical resource for further research on YABBY genes.

## 1. Introduction

The *YABBY* gene family is a group of plant-specific transcription factors that belongs to the zinc finger protein superfamily [1,2,3]. *YABBY* genes play critical roles in plant growth and development [2,3]. In *Arabidopsis thaliana*, six *YABBY* genes have been identified, each playing distinct roles in various developmental stages [1]. *YABBY* genes have been identified and characterized across diverse plant species. In total, 8 *YABBY* genes are found in *Oryza sativa* [4], 7 in *Ananas comosus* [5], 6 in *Platycodon grandifloras* [6], 24 in *Dendrobium chrysotoxum* [7], 7 in *Solanum tuberosum* [8] and 9 in *Melastoma dodecandrum* [9]. Studies of *YABBY* gene family in these species indicate that, beyond regulating growth and development, *YABBY* genes are also involved in various biological processes, including plant hormone responses [10], abiotic stress resistance and light response [11,12]. For example, the overexpression of rice *YABBY1* causes a semi-dwarf phenotype by feedback-regulating gibberellin biosynthesis and metabolism [4]; the overexpression of pineapple *YABBY4* can negatively regulate the salt tolerance of plants [5]. These studies indicate that the *YABBY* gene family has undergone species-specific expansion during evolution. This expansion has driven its functional diversification, which in turn contributes to the adaptation of plants to various environmental challenges.

Cotton is an important economic crop, globally cultivated and valued for its production of natural textile fibers. Cotton fiber is a kind of single cell that differentiates from the epidermis of the ovule. The fiber development can be divided into four distinct and overlapping stages, mainly in terms of days post-anthesis (DPA): cell initiation (−3 to 3 DPA), cell elongation (3 to 20 DPA), secondary cell wall synthesis (20 to 45 DPA) and dehydration maturation (45 to 50 DPA) [13]. The development of fiber has a significant impact on both its yield and quality. Despite the agronomic importance of this process, the *YABBY* gene family in cotton has not been extensively studied, and its specific functions in fiber development remain largely unknown. In this study, RNA-seq data will be employed to investigate the expression profiles of the *YABBY* gene family across different fiber developmental stages. The findings will contribute to a more comprehensive understanding of the underlying mechanisms and support the improvement of cotton yield and quality.

This study conducted a genome-wide identification of the *YABBY* gene family in cotton. We systematically analyzed the physicochemical properties, conserved domains, cis-acting elements, evolutionary trajectory, miRNA–mRNA regulatory networks and expression patterns of the identified genes. The results of this study provide new insights into the evolution and function of *YABBY* in cotton and offer valuable information for future studies on *YABBY* function [14].

## 2. Materials and Methods

### 2.1. Identification of YABBY Gene Family in Gossypium Species

The genome sequence and annotation files of *G. hirsutum* [15], *G. barbadense* [15], *G. arboretum* [16] and *G. raimondii* [17] were obtained from COTTONGEN (http://www.cottongen.org, accessed on 12 March 2025). The *YABBY* protein sequences of the *A. thaliana* and *O. sativa* were used as queries to perform a BLASTP 2.15 [18] search (E-value < 1 × 10^−10^). The hidden Markov model of the *YABBY* domain (PF04690) was used as a query for a homology search with HMMER 3.0 software [19] (E-value < 1 × 10^−10^). The candidate sequences were then submitted to the NCBI CDD (https://www.ncbi.nlm.nih.gov/cdd, accessed on 25 March 2025) to confirm the presence of the *YABBY* domain. Finally, the theoretical isoelectric point and molecular weight of the deduced proteins were predicted using the ExPASy tool [20] (https://web.expasy.org/compute_pi/, accessed on 28 March 2025).

### 2.2. Multiple Sequence Alignment and Phylogenetic Tree Construction

All the identified cotton *YABBY* proteins were aligned using ClustalW [21]. Based on this alignment, a maximum likelihood phylogenetic tree was constructed in MEGA12 [22] with 1000 bootstrap replicates, and subsequently visualized using the iTOL7.2 [23].

### 2.3. Gene Structure, Protein Motif and Cis-Element of Cotton YABBY Gene Family

The exon–intron structures of cotton *YABBY* genes were analyzed using the Gene Structure Display Server [24] (http://gsds.gao-lab.org/, accessed on 2 April 2025), and cis-elements within the 1500 bp promoter region upstream of all genes were predicted using PlantCARE [25]. The conserved motifs of cotton *YABBY* proteins were identified using the MEME suite 5.5.9 [26] (https://meme-suite.org/meme/, accessed on 2 April 2025).

### 2.4. Gene Duplication Analysis of Cotton YABBY Gene Family

Gene duplication events within a cotton genome and across the four Gossypium species (*G. hirsutum*, *G. barbadense*, *G. arboretum* and *G. raimondii*) were analyzed using MCScanX 1.0.0 [27] with default parameters. The collinear relationship was visualized using Circos 0.69-9 [28]. The synonymous substitution (Ks) and non-synonymous substitution (Ka) rates for duplicated gene pairs were calculated using the KaKs_Calculator 2.0 [29]. Divergence times of the duplicated gene pairs were estimated using Formula (1), with the divergence rate λ = 1.5 × 10^−8^ [30].

T = Ks/2λ(1)

### 2.5. Transposable Elements Analysis of Cotton YABBY Gene Family

We detected the transposable elements within 5 kb flanking regions of cotton *YABBY* genes using RepeatMasker 4.1.6 [31] with RepBase; in addition, the abundance and types of the detected transposable elements were analyzed using Perl script.

### 2.6. Prediction of miRNA Targeting YABBY Genes

The CDS sequences of *G. hirsutum YABBY* genes were used to predict the miRNA target sites using psRNATarget (2017 release) [32] with default parameters (https://www.zhaolab.org/psRNATarget/, accessed on 12 April 2025). The resulting miRNA–target interactions were visualized using the R package ggalluvia 0.12.5.

### 2.7. Expression Analysis of Cotton YABBY Genes

RNA-seq data of *G. hirsutum* and *G. barbadense* were downloaded from the NCBI SRA database (http://www.ncbi.nlm.nih.gov/, accessed on 18 April 2025). The SRA data for multiple tissues, different fiber development stages (BioProject: PRJNA490626) and long-day and short-day conditions (BioProject: PRJNA529417) were converted to FASTQ format using the SRAToolkit 3.1.0 [33]. The resulting reads were then aligned to the respective reference genomes using HISAT2 [34], and gene expression levels were quantified in FPKM with Cufflinks 2.1.1 [35]. The expression matrix of the *YABBY* gene family was visualized as a heatmap using the R package of pheatmap 4.4.3.

## 3. Results

### 3.1. Identification of YABBY Gene Family in Cotton

A total of 23, 24, 12 and 37 *YABBY* genes were identified in the genomes of *G. hirsutum*, *G. barbadense*, *G. arboretum* and *G. raimondii*, respectively (Supplementary File S1, Table S1). The number of *YABBY* genes in the diploid *G. arboretum* genome was generally consistent with the number in rice (8) [4]; however, in diploid *G. raimondii*, there are many more *YABBY* genes than in diploid *G. arboretum*.

**Figure 1:**
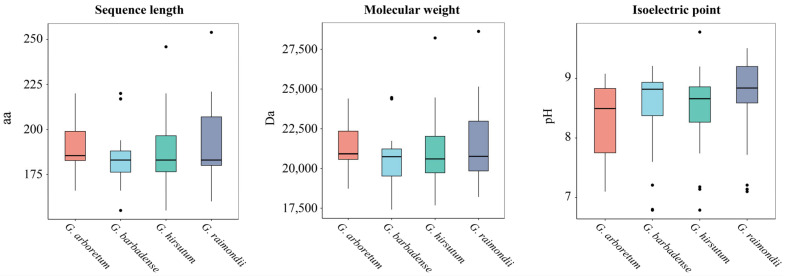
Boxplots of sequence length, molecular weight and isoelectronic point of *YABBY* proteins in *G. arboretum*, *G. barbadense*, *G. hirsutum* and *G. raimondii*.

The physicochemical analysis showed that the length of the identified *YABBY* proteins ranged from 155 to 254 amino acids. The molecular weight of them ranged from 17,422.81 to 28,622.46 Da and the isoelectric point ranged from 6.79 to 9.78 (Figure 1, Supplementary File S1, Table S1).

### 3.2. Phylogenetic Analysis of the YABBY Gene Family

**Figure 2:**
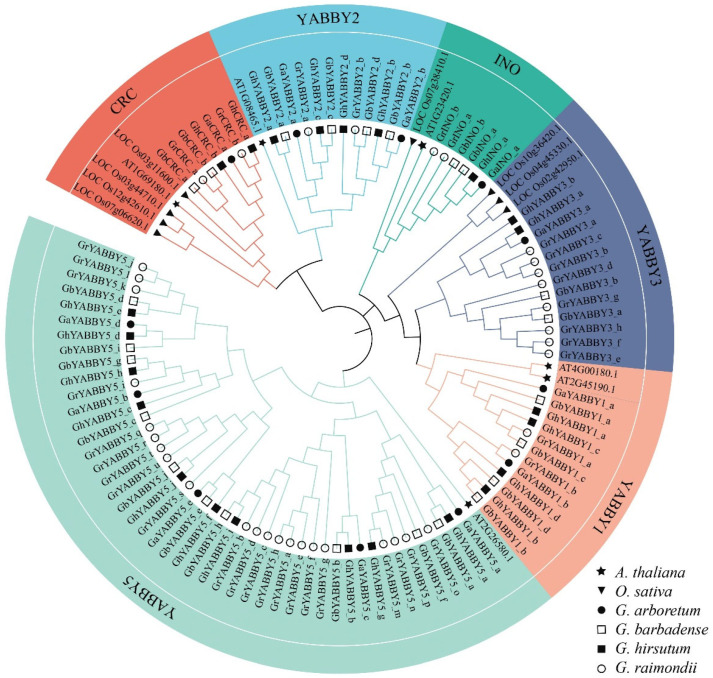
Phylogenetic tree of the cotton *YABBY* gene family.

To investigate the evolutionary relationship of the *YABBY* gene family, a phylogenetic tree was constructed using the maximum likelihood method based on their protein sequences. According to previous studies, the *YABBY* family in *A. thaliana* is divided into six subgroups [1]. In this study, cotton *YABBY* genes were clustered with their closest homologs from *A. thaliana* and were accordingly classified into the same six groups: *YABBY1* (*FIL*), *YABBY2*, *YABBY3*, *YABBY4* (*INO*), *YABBY5* and *CRC*. To facilitate clarity and consistency, cotton *YABBY* genes were renamed with a systematic nomenclature. Each gene identifier begins with a two-letter species code: Gh (*G. hirsutum*), Gb (*G. barbadense*), Ga (*G. arboreum*) and Gr (*G. raimondii*). The subsequent characters represent homology to *A. thaliana YABBY* genes, and the characters after the underscore represent sequential identifiers to distinguish different genes. Among the six groups, *YABBY5* was the largest and included 46 *YABBY* genes, with 5 from *G. arboretum*, 10 from *G. hirsutum*, 10 from *G. barbadense* and 21 from *G. raimondii*. In both groups *YABBY1* and *YABBY2*, each of the diploid species *G. arboreum* and *G. raimondii* contains two *YABBY* genes, while each of the tetraploid species *G. hirsutum* and *G. barbadense* contains four *YABBY* genes. Furthermore, in the group *YABBY3*, the number of *YABBY* genes in *G. hirsutum* increased significantly, whereas the other three cotton species showed almost no amplification. In contrast, the remaining groups exhibited a very small amplification in cotton compared to *A. thaliana* (Figure 2; Supplementary File S1, Table S1). In summary, during evolution, different groups of the cotton *YABBY* gene family experienced varying degrees of expansion, with Gr showing particularly unique amplification.

### 3.3. Gene Structure and Conserved Motif of Cotton YABBY Gene Family

Previous studies have revealed that the *YABBY* gene family contains two highly conserved domains: the N-terminal zinc finger domain and C-terminal *YABBY* domain [1,3]. In the *A. thaliana* CRC protein, the zinc finger domain corresponds to residues 26–53. In this study, a total of two conserved motifs were predicted in cotton *YABBY* proteins: N-terminal Motif 1 and C-terminal Motif 2 (Supplementary File S2, Figure S1). These two motifs are highly conserved and are present in almost all cotton *YABBY* proteins. Additionally, their positions are similar among proteins within the same group. Notably, Motif 1 was absent in two proteins of the *YABBY1* groups in *G. hirsutum* (*GhYABBY1_b* and *GhYABBY1_d*) and *G. barbadense* (*GbYABBY1_d* and *GbYABBY1_b*), respectively, whereas five proteins (*GhINO_a*, *GbINO_a*, *GbINO_b*, *GrINO_a* and *GrINO_b*) of the INO group contained two copies of Motif 1 (Supplementary File S2, Figure S1). Motif 2 was universally present across all cotton *YABBY* proteins.

The exon number of the cotton *YABBY* gene family ranged from 5 to 15, and 85.4% (82/96) of the *YABBY* genes possessed 6~8 exons. The structure of the cotton *YABBY* genes was similar to that of *Melastoma dodecandrum* [9] and *Cucumis sativus* [11], suggesting that the *YABBY* gene structure was relatively conserved.

### 3.4. Key Cis-Element Analysis of Cotton YABBY Gene Family

**Figure 3:**
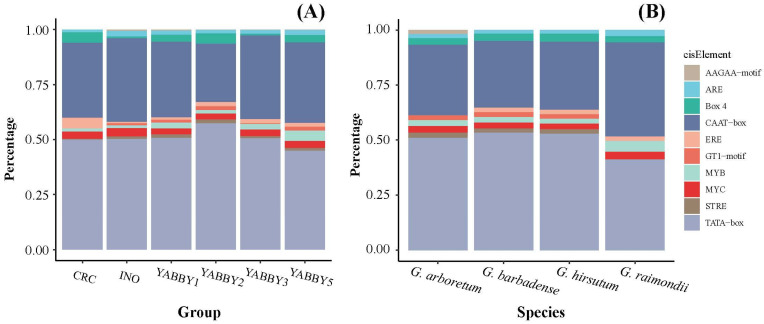
Cis-element composition proportion of cotton *YABBY* gene. (**A**) Different group; (**B**) different species.

The cis-elements within the 1.5 kb upstream promoter region of cotton *YABBY* genes were predicted using PlantCARE. The result indicates that CAAT-box and TATA-box were the most abundant elements, which were common cis-acting elements in promoter regions [24]. After the exclusion of these common elements, cis-elements related to stress responses (MYB, MYC, STRE), light responsiveness (Box 4, GT1-motif) and others were also identified. Furthermore, the proportions of these cis-elements were similar across different species and groups (Figure 3), suggesting that the cis-element composition of *YABBY* genes has been highly conserved since the divergence of cotton species.

### 3.5. Gene Duplication Analysis of Cotton YABBY Gene Family

Gene duplication was the most important cause of gene family expansion during plant evolution. To elucidate the mechanism of *YABBY* gene amplification in cotton, we conducted a collinearity analysis. In *G. arboretum*, 7 out of 12 *YABBY* genes formed 6 duplicated pairs, while, in *G. raimondii*, only 9 out of 37 *YABBY* genes formed 5 duplicated pairs. Additionally, 11 *YABBY* genes from the diploid *G. arboretum* were syntenic with 11 *YABBY* genes from the diploid *G. raimondii*. These results suggest that segmental duplications played a crucial role in the expansion of the *YABBY* gene family in *G. arboreum*. However, despite having a significantly greater number of *YABBY* genes in *G. raimondii*, most of these genes were not generated through segment duplication.

**Figure 4:**
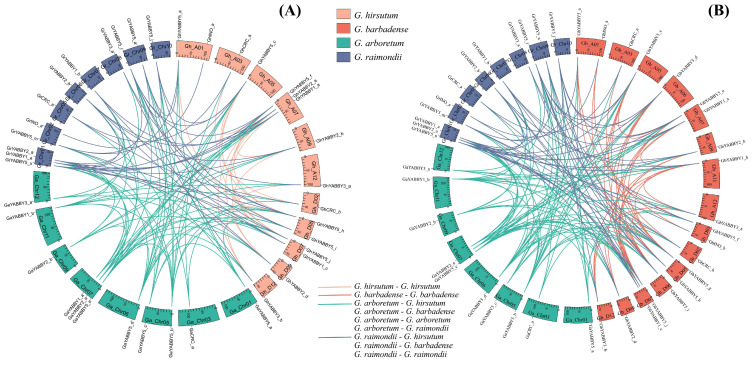
Collinearity analyses of *YABBY* genes in diploid and allotetraploid Gossypium species. (**A**) Diploid *G. arboretum*, *G. raimondii* and allotetraploid *G. hirsutum*. (**B**) Diploid *G. arboretum*, *G. raimondii* and allotetraploid *G. barbadense*.

*G. hirsutum* (AtDt) and *G. barbadense* (AtDt) are typical allotetraploid species, which were derived from the hybridization of two diploid species resembling the ancestors of *G. arboretum* (AA) and *G. raimondii* (DD) [35]. In *G. hirsutum*, 15 of the 23 *YABBY* genes had orthologs in *G. arboreum*, and 16 genes had orthologs in *G. raimondii*; in addition, 15 genes had orthologs in both *G. arboreum* and *G. raimondii*, while 7 genes are lacking orthologs in both *G. arboreum* and *G. raimondii*. In *G. barbadense*, 19 of the 24 *YABBY* genes had orthologs in the *G. arboreum* genome, and 21 genes had orthologs in *G. raimondii*; 19 genes had orthologs in both *G. arboreum* and *G. raimondii*, while only 3 genes are lacking orthologs in both *G. arboreum* and *G. raimondii* (Figure 4).

According to the descriptions of Holub, a chromosomal region within 200 kb containing two or more genes is defined as a tandem duplication event [36]. Two *YABBY* genes (*GrYABBY5_u* and *GrYABBY5_s*) were clustered into a tandem duplication event region on chromosome 01 (NC_026929.1) of *G. raimondii*.

### 3.6. Transposable Element Analysis of Cotton YABBY Gene Family

In addition to segmental duplication and tandem duplication, transposable elements also contribute significantly to the expansion of gene families. In this study, we identified the transposable elements located within 10 kb upstream and downstream of the cotton *YABBY* genes. Our results indicate that 69.57% (16/23), 70.83% (17/24), 75.00% (9/12) and 89.19% (33/37) of *YABBY* genes are close to transposable elements in *G. hirsutum*, *G. barbadense*, *G. arboretum* and *G. raimondii*, respectively. Of these transposable elements, most of them are LTR retrotransposons and DNA transposon (Supplementary File S3, Table S2). These findings suggest that transposable element activity has played an important role in the expansion of the *YABBY* gene family in cotton.

As a diploid cotton species, *G. raimondii* was found to contain 37 *YABBY* genes, a number exceeding those in the allotetraploid species *G. hirsutum* and *G. barbadense*. The transposable element analysis results showed that the number of transposons around the *YABBY* genes of *G. raimondii* is significantly higher than the number in the other three cotton species. These results suggest that transposon activity is an important driver of *YABBY* gene family expansion in *G. raimondii*.

### 3.7. The Selection Pressure and Divergence Time of Cotton YABBY Gene Family

**Figure 5:**
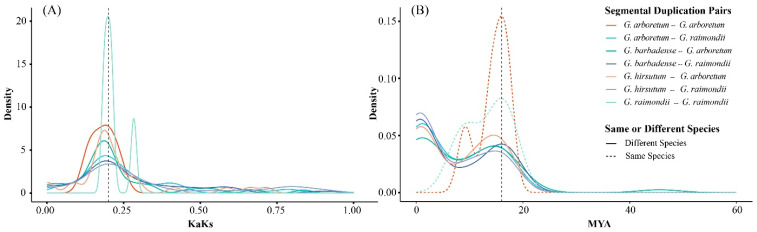
The distribution of Ka/Ks ratio and divergence time of cotton *YABBY* duplication gene pairs. (**A**) The distribution of Ka/Ks ratio; (**B**) the distribution of divergence time.

The ratio of non-synonymous (Ka) to synonymous (Ks) substitutions serves as an indicator of selection pressure on a gene family [37]. To assess the selection pressure on *YABBY* genes, we calculated the Ka/Ks ratios of duplicated gene pairs across different cotton species. The results revealed the that Ka/Ks ratios across different cotton species all ranged from 0.09 to 0.28 (Figure 5), indicating that cotton *YABBY* genes have undergone purifying selection during evolution.

Furthermore, we estimated the divergence time of the cotton *YABBY* gene family using Ks values. The majority of *YABBY* genes in diploid cotton genomes underwent segmental duplications approximately 7 to 21 MYA (Million Years Ago), while duplications between diploid cotton genomes, as well as between diploid and tetraploid genomes, occurred approximately 0 to 22 MYA (Figure 5).

### 3.8. The miRNA Targeting Analysis of G. hirsutum YABBY Genes

**Figure 6:**
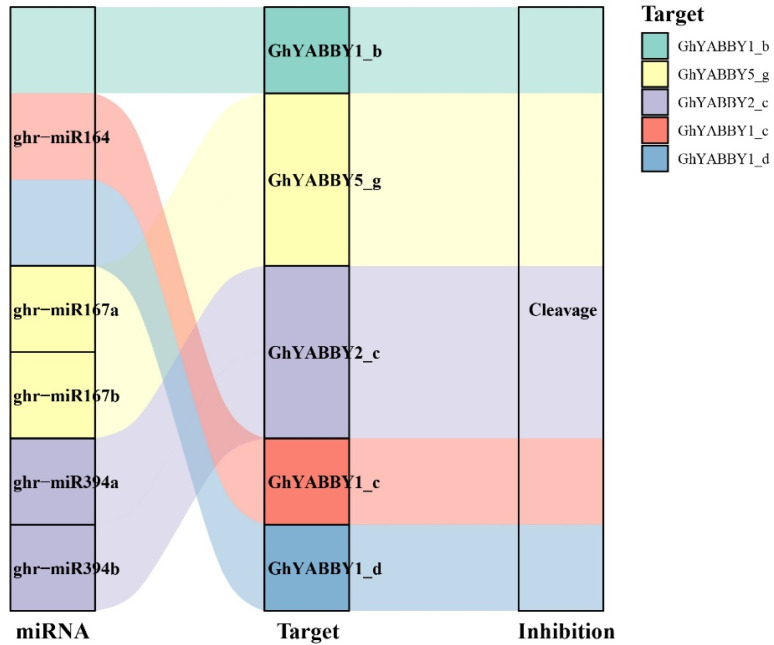
Sankey diagram for the relationships of miRNA-targeting *YABBY* gene transcripts.

MicroRNAs (miRNAs) are crucial players in post-transcriptional regulation. To investigate the miRNA-mediated post-transcriptional regulation of *YABBY* genes in cotton, we identified five miRNAs targeting five *G. hirsutum YABBY* genes. The miRNA ghr-miR164 targeted three YABBY genes from group *YABBY1* (*GhYABBY1_b*, *GhYABBY1_c*, *GhYABBY1_d*). Additionally, *GhYABBY5_g* was targeted by both ghr-miR167a and ghr-miR167b, and *GhYABBY2_c* was targeted by both ghr-miR394a and ghr-miR394b. Our results indicate that all of these miRNAs inhibit the expression of *G. hirsutum YABBY* genes by cleavage (Figure 6).

### 3.9. Expression of Cotton YABBY Genes in Different Tissues

**Figure 7:**
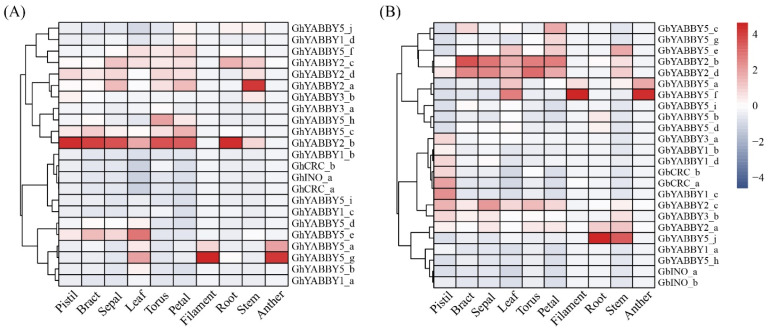
Expression profiles of the cotton *YABBY* genes in different tissues. (**A**) *G. hirsutum*; (**B**) *G. barbadense*.

To investigate the tissue-specific expression profiles of the *YABBY* genes, we analyzed their expressions in the pistil, bract, sepal, leaf, torus, petal, filament, root, stem and anther of both *G. hirsutum* and *G. barbadense*. The genes showed differences in expression among tissues (Figure 7). Specifically, *GhYABBY2_b* of *G. hirsutum* and *GbYABBY2_b* and *GbYABBY2_d* of *G. barbadense* were highly expressed in the pistil, bract, sepal, leaf, torus and petal. In contrast, *GhYABBY5_g* and *GbYABBY5_f* showed the highest expression in the anther and filament. However, most other genes tend to be lowly expressed in the tested tissues. Furthermore, as can be seen from Figure 7, expression profiles varied even among genes within the same group. These data show that genes in the *YABBY2* group are primarily expressed in the pistil, bract, sepal, leaf, torus and petal, while those in the *YABBY5* group are mainly expressed in the anther and filament.

### 3.10. Expression Patterns of Cotton YABBY Genes at Different Stages of Fiber Development

**Figure 8:**
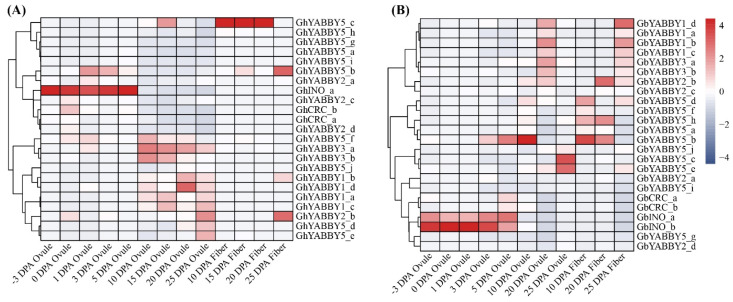
Expression patterns of cotton *YABBY* genes at different stages of fiber development. (**A**) *G. hirsutum*; (**B**) *G. barbadense*.

Cotton fibers are formed by a single ovule outer bead epidermal cell through a specialized process of initial differentiation, elongation, thickening and dehydration to form mature epidermal fibers [13]. To explore the potential roles of *YABBY* genes in fiber development, we investigated their expression patterns at different fiber development stages in allotetraploid cotton. In ovules, *GhINO_a* in *G. hirsutum* and *GbINO_a* and *GbINO_b* in *G. barbadense* exhibited high expression levels from –3 to 5 days post-anthesis (DPA). In addition, three members (*GbYABBY5_b*, *GbYABBY5_c* and *GbYABBY5_e*) of the *YABBY5* group in *G. barbadense*, as well as some genes from the *YABBY1*, *YABBY3* and *YABBY5* groups in *G. hirsutum*, showed high expression levels between 10 and 25 DPA. Compared to the stage of −3 to 5 DPA, the expression level in this stage decreased, but more genes were expressed both in *G. hirsutum* and *G. barbadense*. In fiber, *GhYABBY5_c* in *G. hirsutum* was highly expressed from 10 to 20 DPA, followed by a sharp decline at 25 DPA. In contrast, only a weak expression was detected in *G. barbadense* during this period, involving genes of the *YABBY5*, *YABBY2*, *YABBY3* and *YABBY1* groups (Figure 8). These results suggest that, during the −3 to 5 DPA stage, only genes from group *INO* were highly expressed, whereas in subsequent stages the expression of more genes from other groups increased but remained lower than those in group *INO*. This expression pattern suggests that the *YABBY* gene family may have undergone functional divergence in regulating fiber development.

### 3.11. Expression Patterns of Cotton YABBY Genes in Long- and Short-Day Conditions

**Figure 9:**
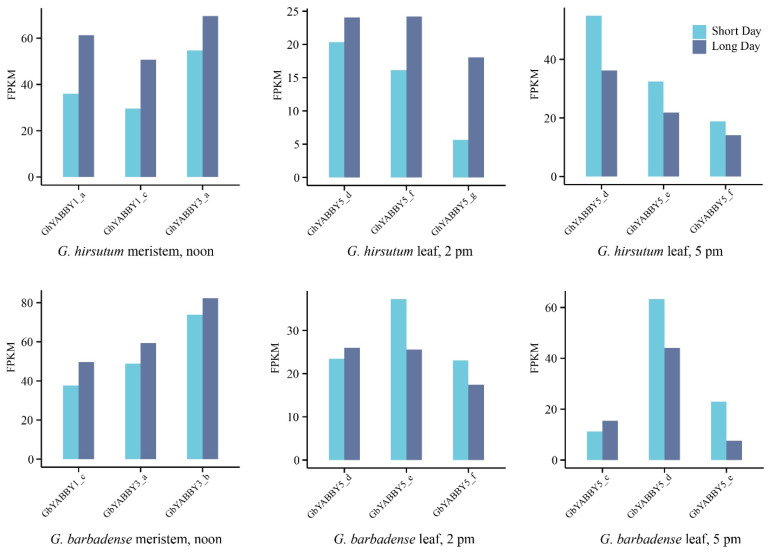
Expression patterns of cotton *YABBY* genes in long- and short-day conditions.

To investigate the molecular function of the *YABBY* family in long-day and short-day conditions, the transcriptome data from the leaf and meristem of *G. hirsutum* and *G. barbadense* were used to calculate the expression of *YABBY* genes. According to NCBI annotations (BioProject: PRJNA529417), leaf samples were collected at 2 pm and 5 pm for long- and short-day conditions, and meristem samples were collected at noon. The analysis of the transcriptome data revealed that *GhYABBY1_a*, *GhYABBY1_c* and *GhYABBY3_a* in *G. hirsutum* and *GbYABBY1_c*, *GbYABBY3_a* and *GbYABBY3_b* in *G. barbadense* were predominantly expressed in the meristem, and all of the expressions were higher in long-day than in short-day conditions (Figure 9). Members of the *YABBY5* group were highly expressed in the leaves of both *G. hirsutum* and *G. barbadense* under long-day and short-day conditions. However, expression patterns diverged among specific genes: for instance, *GhYABBY5_g* and *GhYABBY5_f* exhibited a higher expression under long-day than short-day conditions, whereas the opposite pattern was observed for *GbYABBY5_e* and *GhYABBY5_d* (Figure 9). In general, long-day and short-day conditions mainly influence the expression of genes in the groups *YABBY1* and *YABBY3* and the group *YABBY5*.

## 4. Discussion

The *YABBY* domain-containing gene family has been shown to play a crucial role in diverse aspects of leaf, shoot and flower development [1,3]. Recently, extensive research has been conducted on the *YABBY* genes in different plant species [5,6,7,9]. In this study, we utilized the bioinformatics method to conduct a comprehensive and systematic analysis of the cotton *YABBY* gene family.

The distribution of introns and exons is a typical evolutionary markers of plant gene families [38]. In this study, we found that most *YABBY* genes in the same group contain similar gene structures, implying that they may have similar functions. Nevertheless, divergent gene structures can also be observed within the same group. This may be an important reason why *YABBY* gene family members play different roles in plants. Cis-elements in the promoter region play an important role in gene expression regulation [39]. Our analysis showed that common elements were highly abundant in the promoter of cotton *YABBY* genes. In addition, many other types of cis-elements were found, including elements related to stress response, light responsiveness and others (Figure 3). The result suggests that the cotton *YABBY* gene family was involved in a wide range of biological functions. In addition, the conserved motif analysis revealed that a small number of *YABBY* proteins exhibited variations in their motifs, which indicates that the *YABBY* gene family has undergone divergence during evolution.

Gene duplication serves as a pivotal source of genetic diversity for organisms. Through the processes of gene duplication, followed by mutations and selections, organisms can evolve novel gene functions and traits. It was suggested that the last common ancestor of extant seed plants had only one or two *YABBY* genes [40]. In this study, we identified 23, 24, 12 and 37 *YABBY* genes in *G. hirsutum*, *G. barbadense*, *G. arboreum* and *G. raimondii*, respectively. The number is significantly greater than that found in Arabidopsis [1] and rice [4]. Our results indicated that segment duplication was the primary driver of cotton *YABBY* gene family amplification. Indeed, this is similar to that of other cotton gene families, such as the ALOG [41,42] and ZF-HD families.

Interestingly, the number of *YABBY* genes in diploid *G. raimondii* is nearly three times that of diploid *G. arboreum*. However, our duplication analyses indicated that the number of *YABBY* genes in *G. raimondii* and *G. arboreum* should be similar. To gain deeper insights into this phenomena, we identified the transposable elements flanking *YABBY* genes in the four cotton species. We found that the number of transposable elements located near the *YABBY* genes of *G. raimondii* was significantly higher than that of the other three species. Therefore, it can be concluded that, in addition to segmental duplication, transposon activity has further contributed to the expansion of the *YABBY* gene family in *G. raimondii*.

As tetraploid cotton species (*G. hirsutum* and *G. barbadense*) originated from the hybridization of diploid *G. arboretum* (AA) and *G. raimondii* (DD) [35], the syntenic relationships between tetraploid and diploid cottons is crucial for tracing the origin of the *YABBY* gene family. By comparing syntenic gene pairs of *G. hirsutum*–*G. arboreum*, *G. hirsutum*–*G. raimondii*, *G. barbadense*–*G. arboreum* and *G. barbadense*–*G. raimondii*, we found that tetraploid species have a similar number of syntenic gene pairs in both the *G. arboretum* (AA) and *G. raimondii* (DD) genomes. Studies have shown that the A-derived subgenome was more active than the D-derived subgenome during the evolution of tetraploid cotton [43]. However, this is not the case for *YABBY* genes, underscoring the evolutionary diversity of gene families.

To study the biological functions of cotton *YABBY* genes, the expression profiles were analyzed. The results revealed that genes within the same group often exhibit similar expression patterns, indicating that they are conserved. Meanwhile, not all members within a group share the same expression pattern. These observations, combined with the results of duplication events, suggest that the cotton *YABBY* family has undergone functional diversification after its amplification.

## 5. Conclusions

In this study, we identified 96 *YABBY* genes from four cotton species. Our findings reveal that segmental duplication was the major driver of the family’s expansion. Furthermore, in *G. raimondii*, transposon activity provided an additional force for *YABBY* gene expansion, which accounts for its notably higher gene count. Gene structure, motifs, cis-elements and expression results indicated that the cotton *YABBY* gene family has undergone diversification during evolution. This diversification has consequently led to its diverse biological functions. These findings not only advance our understanding of the *YABBY* gene family but also highlight the potential of these genes as key players for improving cotton fiber yield and quality.

## Data Availability

All data generated or analyzed during this study are included in this published article and its Supplementary Information Files.

## Supplementary Materials

The following supporting information can be downloaded at https://www.mdpi.com/article/10.3390/genes17010064/s1. Supplementary File S1, Table S1: Characteristics of cotton *YABBY* gene family. Supplementary File S2, Figure S1: Phylogenetic relationships, gene structure and conserved motifs of cotton *YABBY* gene family. Supplementary File S3, Table S2: Transposable elements around cotton *YABBY* gene family.
