# Supplementary figures and images for: Genomic Identification and Characterization of the Cotton YABBY Gene Family
Source: Genes (Basel). 2026 Jan 6;17(1):64. doi: 10.3390/genes17010064 (PMC12841406; doi:10.3390/genes17010064)

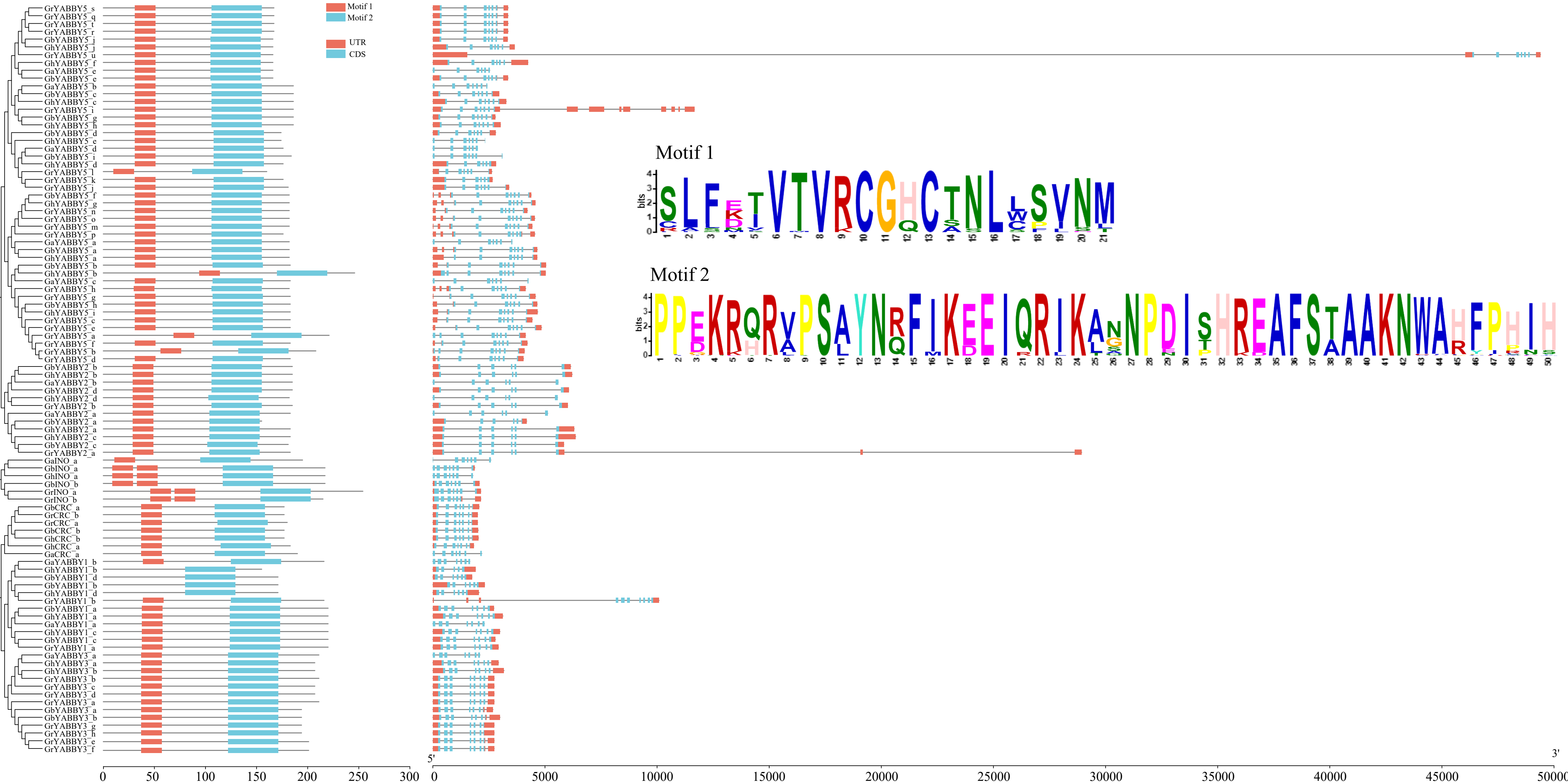

Supplement: Supplementary file 1 [file genes-17-00064-s001.zip › Supplementary File S2 Figure S1.pdf]
